# SALL4 as a transcriptional and epigenetic regulator in normal and leukemic hematopoiesis

**DOI:** 10.1186/s40364-017-0115-6

**Published:** 2018-01-03

**Authors:** Jianchang Yang

**Affiliations:** Department of Surgery and Medicine, Baylor College of Medicine, Houston, TX 77030 USA

**Keywords:** Pluripotency, Hematopoietic stem and progenitor cells, Apoptosis, Transgenic, Wnt/β-catenin signaling, Mixed-lineage leukemia, Chromatin modification

## Abstract

In recent years, there has been substantial progress in our knowledge of the molecular pathways by which stem cell factor SALL4 regulates the embryonic stem cell (ESC) properties, developmental events, and human cancers. This review summarizes recent advances in the biology of SALL4 with a focus on its regulatory functions in normal and leukemic hematopoiesis. In the normal hematopoietic system, expression of SALL4 is mainly enriched in the bone marrow hematopoietic stem/progenitor cells (HSCs/HPCs), but is rapidly silenced following lineage differentiation. In hematopoietic malignancies, however, SALL4 expression is abnormally re-activated and linked with deteriorated disease status in patients. Further, SALL4 activation participates in the pathogenesis of tumor initiation and disease progression. Thus, a better understanding of SALL4’s biologic functions and mechanisms will facilitate development of advanced targeted anti-leukemia approaches in future.

## Background

SALL4 is one of four human homologues (SALL1, 2, 3, and 4) of the Drosophila region-specific homeotic gene *spalt* (*sal*) [1–4]. SALL4 encodes multiple Cys2His2 zinc finger (C2H2-ZF) domain-containing transcription factor that either activates or represses gene transcription depending on cell context. In mammals, expression of SALL4 has been primarily detected in ESCs and adult tissue “stem-like” cell populations, where it acts as a core controller regulating cell “stemness” in developmental events and also in tumor growth [5–9]. To date, aberrant SALL4 expression in humans has been observed in over 10 types of solid tumors and in several common types of leukemia (see review [8–11]), and SALL4 has been considered a biomarker for the diseases. In addition, studies suggest that SALL4 may be a useful therapeutic target in combating human leukemia in clinic [10, 11]. For these reasons, it will be important to understand, how SALL4 as a stem cell factor exerts its effects in different cell contexts, and how we can effectively translate our gained knowledge into treatment breakthroughs in future.

## Sall4 stem cell gene

The stem cell factor SALL4 is required for ESC pluripotency and for early embryonic development. It is an essential component of the ‘stemness’ regulatory circuit involving OCT4, SOX2, NANOG and other factors in maintaining ESC self-renewal and pluripotency [12–17]. In ESCs, a well-controlled SALL4/OCT4 transcription regulatory loop balances proper expression dosage of SALL4 and OCT4; and like OCT4, reduction of SALL4 results in re-specification of ESCs to the trophoblast lineage [18–20]. SALL4 is also a critical regulator in reprogramming of somatic cells to pluripotency. Shu et al. recently reported that GATA family members GATA4 and GATA6 can substitute for OCT4 in mouse somatic reprogramming, and identified SALL4 as a major target gene of the GATA members, serving as a bridge linking the lineage-specifying GATA family to the pluripotency circuit [21]. In fact, ectopic expression of SALL4, NANOG, ESRRB, and LIN28 [22], or the combination of SALL4, SALL1, UTF1, NANOG and MYC [23] in embryonic fibroblasts reprogram them into induced pluripotent stem cells (iPSCs) in the absence of OCT4.

In murine development, significant zygotic SALL4 transcription occurs at as early as the 4-cell stage. At the blastocyst stage, SALL4 expression becomes enriched in the inner cell mass (ICM) and the trophectoderm [12, 24–26]. Reduction of SALL4 in oocytes and ESCs results in early embryo defects, and disruption of both *Sall4* alleles causes embryonic lethality during peri-implantation [27–29]. SALL4 is also expressed in extraembryonic endoderm (XEN) cells, where it participates in cell fate decision by simultaneously activating key pluripotency maintaining factors and silencing endoderm lineage-associated factors such as GATA6, GATA4, and SOX17 [30, 31]. During subsequent stages, heterozygous disruption of *Sall4* allele leads to multi-organ malformations including limb and heart defects, which model human disease [27, 29]. It has been reported that TBX5, a gene encoding a T-box transcription factor, regulates SALL4 expression in the developing forelimb and heart, and interacts with SALL4 to synergistically regulate downstream gene expression [28, 29, 32].

In both humans and mice, SALL4 proteins exist at least in three isoforms termed A, B and C, with A and B isoforms being the most studied [32–36]. In ESCs, the SALL4 proteins are sequestered into the nuclear foci and bind to heterochromatin, where they participate in chromatin structure remodeling during transcription repression [37, 38]. SALL4 has also been shown to interact with the histone H3 lysine 36 (H3K36me3)-specific methyltransferase Wolf-Hirschhorn syndrome candidate 1 (WHSC1), which affects histone modification and thus regulate the expression of their target genes [39–41].

## Functions of Sall4 and its regulated networks in normal hematopoiesis

Given SALL4’s prominent roles in ESCs, development, and its specific expression patterns in the hematopoietic system, our research group previously investigated SALL4’s functions in the self-renewal of HSCs/HPCs. We demonstrated that the SALL4 isoforms are robust stimulators for human CD34+ HSCs/HPCs expansion [42, 43]. Of note, we reported that the SALL4-driven ex vivo expansion of HSCs/HPCs is dependent on excessive special cytokines, and does not affect mature colony formation in colony-forming unit (CFU) assays [42]. In another study, Tatetsu et al. report that ex vivo culture of mobilized peripheral blood CD34+ cells with histone deacetylase (HDAC) inhibitors leads to expansion of a CD34+ CD90+ population, and SALL4 is identified as a key transcription factor responsible for the process [44]. In mouse model studies, forced overexpression of SALL4 in Lineage^−^ Sca-1^+^c-Kit^+^ (LSK) bone marrow (BM) cells likewise leads to sustained cell proliferation, as well as enhanced marrow-repopulating potential [45]. Transcripts assays showed that the increased HSC/HPC growth was associated with upregulation of multiple HSC regulatory genes including HOXB4, NOTCH1, BMI1, RUNX1, CMYC, MEIS1 and NF-YA [45]. Further, in a myeloid progenitor cell line (32D cells), overexpression of SALL4 blocked granulocyte-colony stimulating factor (G-CSF)-induced granulocytic differentiation, and permitted expansion of undifferentiated cells in the presence of defined cytokines [42, 45]. Thus, the SALL4 isoforms stimulate HSC/HPC proliferation by activating important self-renewal regulators and simultaneously inhibiting differentiation. Recently, Mossahebi-Mohammadi et al. reported that the CD133^+^ umbilical cord blood HSCs/HPCs are also efficiently expanded following SALL4 lentivirus transduction, and the SALL4-expanded CD133^+^ cells retain self-renewal and differentiation capacities with no chromosomal aberrations [46]. In another study, it is further reported that SALL4 in CD34+ cells was downregulated by microRNAs miR-15b and miR-219-5p, and inhibition of miR-15b, −which activates SALL4 expression, significantly increased the number of CD34^+^ HSCs/HPCs in culture [47].

In elucidating the SALL4 regulated networks, Gao et al. sorted human CD34+ BM cells and performed chromatin immunoprecipitation followed by microarray hybridization (ChIP-on-chip), together with gene expression assays [48]. These works identified that CD34, RUNX1, HOXA9, and PTEN are SALL4-directed target genes. In particular, HOXA9 is being characterized as a major SALL4 target in hematopoiesis. Moreover, down-regulation of SALL4 or HOXA9 expression in CD34+ cells results in similar effects, i.e., reduced in vitro myeloid colony formation and impaired in vivo engraftment [48]. In another study [49], the polycomb complex protein BMI-1 as a critical SALL4 downstream target has been documented. Chromatin immunoprecipitation coupled with quantitative PCR (ChIP-qPCR) in 32D cells reveals that SALL4 binds to a specific region of *Bmi-1* gene promoter, and heterozygous disruption of *Sall4* allele significantly reduced BMI-1 expression in BM cells. Further, in transgenic mice that constitutively overexpress human SALL4B, there is up-regulated expression of BMI-1, and the levels of BMI-1 in these mice increase as they progress from normal to preleukemic (myelodysplastic syndrome [MDS]) and leukemic (acute myeloid leukemia [AML]) stages.

Very recently, SALL4’s functions in normal hematopoiesis have been further explored using conditional gene targeting approaches [50]. Surprisingly, wild type *Sall4*^*f/f*^/CreER^T2^ mice that are treated with tamoxifen, or Vav-Cre-mediated (hematopoietic-specific) *Sall4*^−/−^ mice are all healthy and display no significant hematopoietic defects, which is in contrast to previous human CD34+ cell studies. Reasons for this discrepancy have not been fully addressed. To be noted however, some genes may exert aberrant functions only when cells encounter transplantation or replicative stress (see review [51]), and some Vav-Cre knockout models may demonstrate hematopoietic defects at late stages [52]. Thus it may be necessary to perform serial transplantation and/or stress induction (such as 5-fluorouracil injury) assays with SALL4-deficient cells to fully clarify SALL4 effect and mechanisms in maintaining normal HSCs.

## Functions of Sall4 and its regulated networks in leukemia

### SALL4 as a diagnostic marker in human leukemia

Aberrant expression of SALL4 has been detected in MDS patients and its expression levels are correlated with disease progression [34, 53]. Also, SALL4 and BMI-1 share a similar expression pattern in the patients with both expressions increase in high-grade morphology and high International Prognostic Scoring System (IPSS) score cases [53]. In addition, higher SALL4 protein levels are associated with the complex karyotype (equal to or more than three aberrant karyotypes), and SALL4 appears to be involved in the DNA damage response in patients [54], as supported by their roles in the ESC system [38]. In AML cases, immunohistochemistry staining reveals that SALL4 proteins are present in nearly all the patient samples that are examined (*n* = 81, subtypes M1 to M5, the French-American-British [FAB] classification) [34], and SALL4 expression in patients with complex karyotype is significantly higher than that in MDS patients with normal karyotype [54]. Similarly, variable transcript levels of SALL4A and SALL4B expression are reported in pediatric AML patients [55]. In other leukemia cases, aberrant SALL4 expression has been reported in ALK positive anaplastic large cell lymphoma (ALK^+^ ALCL) [56], B cell acute lymphocytic leukemia (B-ALL), −most prominently in B-ALL patients with TEL-AML1 translocation, which is the most common genetic abnormality in pediatric B-ALL [57, 58]. SALL4 expression is also detected in patient samples from blastic stage of chronic myeloid leukemia (CML), as contrast to the chronic phase, and CML patients who have achieved complete remission or those who have tyrosine kinase inhibitor resistance [59–61]. Lastly, constitutive SALL4 expression has been documented in various human myeloid and lymphoid leukemia cell lines including KG1a (AML-M0), KASUMI-1 (AML-M2), HL-60 (AML- M2/M3), NB-4 (AML M3), THP1 (AML-M5), TEX (FUS/TLS-ERG oncogene immortalized AML), K562 (CML/AML M6), RPMI-8226 (myeloma), LAMA84 (CML-acute phase), BV173 (B-ALL), REH (ALL), NALM6 (ALL), 697 (pre-B ALL), BLIN-1 (pre-B ALL) and JURKAT (T cell leukemia) [34, 58, 61–65].

Interestingly, the SALL4 expression appears to be selectively enriched in leukemia side-population (SP) cells (defined by low Hoechst 33,342 blue/red fluorescence intensity), −a fraction specifically involved in cancer initiation and drug resistance [66], which supports the SALL4 expression features observed in above clinical cases, and suggests a role of SALL4 in maintaining the leukemia “initiating or stem” cell (LIC/LSC) populations.

### Leukemogenesis induction by SALL4B in a transgenic model

In 2006, our research group reported the first SALL4-mediated leukemogenesis model [34]. We investigated transgenic mice that overexpress human SALL4A or SALL4B proteins, controlled by a universal CMV promoter. All the *SALL4B* mice from 6 founders developed MDS-like features at 2 months of age, and 9 of them (53%) progressed to AML. By contrast, none of the SALL4A mouse exhibits leukemia formation during the test period [34, 67]. These groups of studies suggest that SALL4B, but not SALL4A, is a novel oncogene in inducing leukemogenesis. As an adding note, however, while forced overexpression of either SALL4A or SALL4B isoform fails to transform primary BM cells, nor induces leukemia formation in transplanted mice [43, 67, 68], one may deduce that in the *SALL4B* transgenic mice, an abnormal “niche” resulting from aberrant SALL4B expression in various mouse tissues throughout the mouse development may play a role, and/or SALL4B may bear oncogenic potential during the dysregulated hematopoiesis. Further in-depth studies are needed to elucidate the associated mechanisms.

### Role of SALL4 in mixed lineage leukemia (MLL)- rearranged (MLL-r) leukemogenesis

Considering the strong association of SALL4 expression with tumor initiation and leukemia survival, our group recently explored its effects in the pathogenesis of leukemia mediated by MLL-AF9, −one of the most common MLL-r oncoproteins found in leukemia patients [69–71]. MLL-r leukemias have been very challenging in therapy and associated with poor outcomes [70, 72, 73]. Further, a previous study has shown that the MLL wild type protein physically interacts with SALL4 in regulating HOXA9 expression [74]. We report that SALL4 expression is essentially required for MLL-AF9-mediated myeloid transformation in primary BM cells. Cre recombinase or shRNA mediated SALL4 knockdown in MLL-AF9-transformed cells induced apoptotic death and cell cycle arrest at G1. Consistently, disruption of both *Sall4* alleles in transplanted mouse models completely prevented leukemia initiation and also attenuated pre-existing disease progression [50]. These studies support that the SALL4 pathway may not only be used as a useful biomarker or therapeutic target, proper inhibition of the SALL4 pathway may also effectively prevent disease initiation in patients at early stages.

### SALL4 as a therapeutic target in human leukemic cells

SALL4 as a key regulator of survival has been widely documented in different subtypes of leukemias and in various human cancers [8, 10, 54, 75, 76]. In these malignancies, downregulation of SALL4 prominently causes increased apoptosis and cell cycle arrest [50, 62, 75–78]. In AMLs, aberrant SALL4 has also been shown to block all-trans retinoic acid (ATRA)-mediated myeloid differentiation in both ATRA-sensitive and ATRA-resistant AML subtypes [62]. Thus, these studies support that SALL4 maintains leukemic growth by protecting their proper proliferation and also inhibiting their differentiation. Not surprisingly, in recent years, SALL4-targeted anti-leukemia strategies have gained increasing interest and been elaborately explored (see review [11]). In a study by Gao et al., a SALL4- derived peptide blocking its protein-protein interaction with the nucleosome remodeling and histone deacetylation (NuRD) complex results in notable leukemic cell death, but causes no cytotoxic effects on normal CD34+ HSCs/HPCs [64]. Gao et al., also reported that SALL4 knockdown in combination with BCL-2 inhibitor treatment increased the apoptotic AML cells to 2 to 3 fold as compared to cells treated with each alone [77]. Our research group reported that while SALL4 and its interacting epigenetic factor LSD1 inhibited ATRA-induced granulocytic differentiation, co-inhibition of SALL4, LSD1, plus ATRA treatment severely disrupted ATRA-resistant AML cell growth, and blocked HL60 AML xenograft tumors by ~91%, while the treated mice exhibit no signs of illness [62, 79]. Considering that *Sall4* depletion in mice minimally affects adult hematopoiesis [50], these separate works collectively suggest that the SALL4 regulation-targeted approaches, which induce leukemia apoptosis and differentiation but spare normal cells, likely represent clinically effective and novel anti-leukemia strategies.

### SALL4 regulated pathways in leukemia

ChIP-on-chip assays with a promyelocytic leukemic cell line, NB4 reveal that SALL4-binding genes are involved in more than 30 different signaling pathways [75]. Most prominent of these pathways are WNT/β-catenin, apoptosis, NOTCH signaling, the polycomb complex protein BMI-1, PTEN, and nuclear factor-kB. Further, SALL4 expression in these cells represses important apoptosis-inducing genes (*CARD9, CARD11, CYCS, LTA, TNF, TP53, PTEN*) but promotes apoptosis suppressor genes (*BMI-1, BCL2, DAD1, TEGT, XIAP*). In *SALL4B* transgenic mouse studies, the SALL4A and SALL4B isoforms have been found to bind β-catenin protein, and interaction of these factors synergistically activates the WNT/β-catenin pathway, −which plays critical roles in controlling LSC self-renewal [34, 80–82]. The interaction domain between SALL4 and β-catenin was not determined. However, it was deduced that the C-terminal portion of SALL4 may play a role, based on binding data between SALL1 and β-catenin [8, 83]. Intriguingly, in a recent study by Kode et al., transgenic activation of a mutated *β-catenin* allele in murine osteoblasts, −one of the key HSC/HPC niches in BM [84, 85], induced MDS and AML in mice at very early ages via dysregulated NOTCH ligand JAG1 [86–88]. Since *SALL4B* transgenic mice also develop MDS/AML, and SALL4B-overexpressed BM cells do not induce leukemia formation in transplanted mice, an interesting question will be in the *SALLB* mouse, if/how SALL4B potentially activates β-catenin signaling, which synergistically dysregulate the HSC/HPC osteoblastic niche and thus promote leukemogenesis. Further detailed transgenic studies are required to address this probability.

ChIP assays with sequencing (ChIP-Seq) assays with mouse MLL-AF9 transformed leukemic cells are also performed [50], which identified that SALL4 binds to 451 genes including MLL-AF9 targets (*Meis1, Hoxa9*), MLL-r leukemia-related genes (*Cebpα, Id2, Elf1, Evl, Flt3, Nf1, Tal1, Tcf7l1, Nkx2–3*), HOX factors (*Hoxa-9*, *−10, −11, −13*), Notch ligand *Jag2*, and Wnt/β-catenin regulator *Wnt7b*. Further, loss of SALL4 downregulated the expression levels of *Bmi1, cMyb, Runx1, Meis1*, and HOX factors. In the same MLL-AF9 transformed cells, mRNA microarrays assays following early *Sall4* deletion identified upregulated genes including cell cycle inhibitors *Cdkn1a* (*p21*), *Trp53inp1*; HSC/HPC colony-forming repressor *Slfn2*; and hematopoietic differentiation markers *Col5a1, Fyb, Irf8* and *Pira6*. In contrast, downregulated genes included multiple TGFβ signaling components *Tgfβ2, Tgfβ3* and *Tgfβr3*; genes related to chemo-resistance or leukemia aggressiveness such as *Thbs1*, *Tgm2*, *Ambp*, and the AF9 regulator *Sgk1, −*which negatively regulates the DOT1A-AF9 complex-mediated transcriptional repression [50, 89]. Taken together, these studies suggest that SALL4 may regulate MLL-AF9 mediated leukemogenesis via multiple HSC/HPC- and LSC-related signaling pathways.

To date, upstream regulators of SALL4 expression in leukemia remain poorly understood, although OCT4, GATA4, GATA6, STAT3, TBX5, the WNT/β-catenin signaling factors, and micorRNAs such as miR-98, miR-33b and miR-294 are reported in other cell systems [8–10, 76, 90, 91]. Of note, DNA methylation modifications of the *SALL4* promoter may play a role, −studies using B-ALL cell lines and patient samples detected hypomethylation of the *SALL4* CpG islands spanning the exon1-intron1 region [57], which has also been observed in MDS and AML patients, and this hypomethylation correlates with high SALL4 expression [92, 93]. Some other studies have documented SALL4-repressing compounds, such as apigenin, matrine, indole and its derivative 2-(1-((2, 4-Aril)imino)-2,2,2-trifluoroethyl) phenyl-1H Indole-3- carbaldehyde (TFPHC) are documented [10, 94]. Such works suggest that the SALL4 regulatory pathway may be potentially disrupted by clinically applicable drugs, and more such related ongoing works are expected to promote translation of cutting-edge SALL4 knowledge into clinical practice in future.

## Epigenetic mechanisms involved in Sall4’s regulatory functions in normal and leukemic hematopoiesis

Multiple chromatin modification regulators have been identified involving in SALL4’s regulatory functions. So far the reported SALL4-interacting epigenetic factors include: DNA methyltransferases DNMT-1, −3A, -3B, −3 L, methyl-CpG-binding domain 2 protein (MBD2) [95]; NuRD complex that contains histone deacetylases HDAC1/2 [64, 96]; H3K4 methyltransferase MLL1 [74, 97]; H3K79 methyltransferase DOT1L [50, 98]; H3K36 methyltransferase WHSC1 [39, 99], and lysine-specific histone demethylase LSD1/KDM1A [50, 62, 79]. All of these are critical regulators in normal blood development and are frequent targets for dysregulation in hematological malignancies [100–103]. Of note, the SALL4 proteins seem to interact with these epigenetic factors at different sites. For example, while the amino-terminal 174 amino acid sequence of SALL4 is critical for SALL4-DNMT1 or -HDAC interaction, it seems less relevant to SALL4-LSD1 interaction. This is important in designing protein-protein interaction based anti-SALL4 strategies. Noteworthily, clinical epigenetic remedies inhibiting such SALL4-interacting epigenetic factors have been shown effective in treating leukemia [104–108]. Indeed, in MLL-AF9-mediated mouse AML studies, inhibition of either SALL4, DNMT1, LSD1, or DOT1L completely blocked leukemia initiation and significantly delayed disease progression in vivo [109–111].

By dynamically recruiting each specific epigenetic factor, SALL4 expression can directly affect DNA and histone methylation/acetylation status at important genes that control hematopoietic differentiation, apoptosis, tumor induction or suppression. For example, in NB4 AML cells transduced with lentiviral SALL4, there was an overall increased percentage of DNA methylation (a range of 1.2 to 2-fold) at various CpG sites of tumor suppression gene *PTEN* promoter, which co-relates with a down-regulated gene transcription [95]. In mouse BM LSK cells, overexpression of the SALL4 isoforms also induces increased percentage of methylation (1.2 to 6- fold) at the CpG sites of early B-cell factor 1 (*Ebf1*) promoter, as well as the *Sall4* gene promoter itself, which facilitates an undifferentiated cellular status [95]. Similarly, SALL4 overexpression or Cre-induced *Sall4* gene deletion affected LSD1 binding and altered H3K4me2 levels at the promoter regions of tumor necrosis factor (*Tnf*) and differentiation-related genes *Ebf1*, *Gata1* (up to ~300 and ~700 fold changes), which are associated with relevantly altered gene transcription levels [79]. Further, in 32D myeloid progenitor cells following lentiviral SALL4 transduction, the H3K4me3 and H3K79me2/3 levels at SALL4-occupied regions of *Bmi1* promoter were increased [49]. Additionally, expression levels of SALL4 protein affect H3K4me3 and H3K79me3 amounts at promoter regions of *Meis1* and multiple HOX family genes in normal or leukemic BM cells, as we and others reported [48, 50, 74]. Also, in the functional study by Gao et al., while SALL4 interacts with the HDAC complex, and silences *PTEN* promoter via reduced acetylation of histone H3 at its binding sites, the SALL4-derived peptide blocks this interaction and leads to reactivated PTEN expression, which induces leukemia cell death that can be rescued by a PTEN-specific inhibitor [64, 96].

Evidence suggests that SALL4 may compensatively recruit different epigenetic regulators. For example, LSD1 has been shown to contribute to global DNA methylation via stabilizing DNMT1 [112], while in the absence of LSD1 proteins, SALL4 seems to compensatively recruit DNMT1 or DNMT3L to downstream genes (such as *EBF1*, *TNF*) and modulate their expression [79]. Also, co-inhibition of DNMTs and HDACs can synergistically block SALL4’s regulatory effects in cultured cells, which induces differentiation and cell growth arrest in human leukemia [113]. In addition, LSD1 has been shown to interact with the SALL4/CDX4 (a master regulator of the HOX genes) circuit and affect granulocytic/erythroid maturation [114, 115]. SALL4 also appears to confer cell resistance to DNA double-stranded break (DSB)-induced cytotoxicity by collaborating with HDAC-1 and -2 [38, 116].

**Fig. 1:**
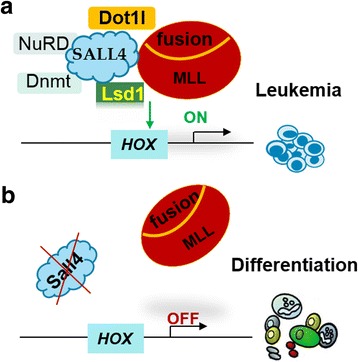
A tentative model for SALL4-regulated epigenetic mechanism in MLL-r leukemia. **a** Through interacting with MLL fusion oncoprotein, DOT1L, LSD1 and other epigenetic factors, SALL4 may act as a “guide” in modulating the expression levels of specific downstream gene targets. **b** SALL4-targetd strategies can disrupt MLL fusion oncoprotein function via dysregulated recruitment of essential epigenetic factors

It is important to note that SALL4’s regulation of specific epigenetic modification programs is strictly dependent on the cellular contexts. Analysis of the ChIP-on-chip data from NB4 AML cells and normal CD34+ HSCs/HPCs reveals distinct SALL4 binding patterns between these two cell types, which reflect a cell type-specific epigenetic signature and SALL4 function [48, 75]. This finding is supported by ChIP-Seq data analysis of ESCs and extra-embryonic endoderm cell studies [30]. In ESCs, the SALL4-bound genomic loci are largely enriched for activating marker H3K4me3, which indicates an association of SALL4 with non-repressed genes. In XEN cells, however, the SALL4-binding loci display significantly less H3K4me3 enrichment. Instead, over 60% of these regions are either accompanied with H3K27me3 or lacking both H3K4me3 and H3K27me3, −the “epi-markers” frequently associated with gene repression. In our MLL-AF9 leukemia model studies [50], SALL4 appears to recruit both DOT1L and LSD1 to specific downstream target genes and modulate their H3K79me2/3 and H3K4me3 levels, thereby maintaining proper gene expression and leukemic survival (see Fig. 1). As reported in previous studies, however, some non-MLL-r human AMLs may not rely on DOT1L-regulated H3K79 methylation, and DOT1L recruitment to MLL-AF9 is further associated with the level of leukemic transformation [117–120]. Thus, we may anticipate that in normal hematopoiesis and such different subtypes of human leukemias, SALL4 should differentially interact with such epigenetic factors during regulation of gene expression, thereby exerting a disease/subtype–dependent regulatory effect. In-depth functional and epigenetic studies are required to prove this assumption.

## Conclusions

This review summarized recent advances in the biology of the stem cell factor SALL4 with a focus on its regulatory functions in normal and leukemic hematopoiesis. In recent years, there have been gains in our understanding of SALL4-regulated molecular mechanisms and SALL4-targeted strategy in killing tumor cells. Understanding how SALL4 mechanisms maintain normal HSCs/HPCs vs. leukemic cells will facilitate development of newer, more efficient anti-leukemia strategies.

## Abbreviations

ALK+ ALCL: ALK positive anaplastic large cell lymphoma
AML: Acute myeloid leukemia
ATRA: All-trans retinoic acid
B-ALL: B cell acute 112 lymphocytic leukemia
BM: Bone marrow
C2H2-ZF: Cys2His2 zinc finger
CFU: Colony-forming unit
ChIP-on-chip: Chromatin immunoprecipitation followed by microarray hybridization
ChIP-PCR: Chromatin immunoprecipitation coupled with quantitative PCR
ChIP-Seq: ChIP assays with sequencing
CML: Chronic myeloid leukemia
Ebf1: Early 229 B-cell factor 1
ESC: Embryonic stem cell
FAB: The French-109 American-British classification
G-CSF: Granulocyte-colony stimulating factor
HDAC: Histone deacetylase
HSPs/HPCs: Hematopoietic stem/progenitor cells
ICM: Inner cell mass
iPSCs: Induced pluripotent stem cells
IPSS: International Prognostic Scoring System
LIC/LSC: Leukemia “initiating or stem” cell
LSK: Lineage- Sca-1+ c-kit+
MBD2: Methyl-CpG-binding domain 2 protein
MDS: Myelodysplastic syndrome
MLL-r: Mixed lineage leukemia (MLL)-rearranged
NuRD: Nucleosome remodeling and deacetylase
SP: Side-population
TFPHC: 2-(1-((2, 4-Aril)imino)-205 2,2,2-trifluoroethyl) phenyl-1H Indole-3- carbaldehyde
Tnf: Tumor necrosis factor
WHSC1: Wolf-Hirschhorn syndrome candidate 1
XEN: Extraembryonic endoderm
